# Composition, structure and tensile biomechanical properties of equine articular cartilage during growth and maturation

**DOI:** 10.1038/s41598-018-29655-5

**Published:** 2018-07-27

**Authors:** J. Oinas, A. P. Ronkainen, L. Rieppo, M. A. J. Finnilä, J. T. Iivarinen, P. R. van Weeren, H. J. Helminen, P. A. J. Brama, R. K. Korhonen, S. Saarakkala

**Affiliations:** 1 Research Unit of Medical Imaging, Physics and Technology, Faculty of Medicine, University of Oulu, Oulu, Finland; 2 Medical Research Center, University of Oulu and Oulu University Hospital, Oulu, Finland; 3 Department of Applied Physics, University of Eastern Finland, Kuopio, Finland; 4 Institute of Biomedicine, University of Eastern Finland, Kuopio, Finland; 5 Department of Equine Sciences, University of Utrecht, Utrecht, Netherlands; 6 Veterinary Clinical Sciences, School of Veterinary Medicine, University College Dublin, Dublin, Ireland; 7 Department of Diagnostic Radiology, Oulu University Hospital, Oulu, Finland

## Abstract

Articular cartilage undergoes structural and biochemical changes during maturation, but the knowledge on how these changes relate to articular cartilage function at different stages of maturation is lacking. Equine articular cartilage samples of four different maturation levels (newborn, 5-month-old, 11-month-old and adult) were collected (*N* = 25). Biomechanical tensile testing, Fourier transform infrared microspectroscopy (FTIR-MS) and polarized light microscopy were used to study the tensile, biochemical and structural properties of articular cartilage, respectively. The tensile modulus was highest and the breaking energy lowest in the newborn group. The collagen and the proteoglycan contents increased with age. The collagen orientation developed with age into an arcade-like orientation. The collagen content, proteoglycan content, and collagen orientation were important predictors of the tensile modulus (*p* < 0.05 in multivariable regression) and correlated significantly also with the breaking energy (*p* < 0.05 in multivariable regression). Partial least squares regression analysis of FTIR-MS data provided accurate predictions for the tensile modulus (*r* = 0.79) and the breaking energy (*r* = 0.65). To conclude, the composition and structure of equine articular cartilage undergoes changes with depth that alter functional properties during maturation, with the typical properties of mature tissue reached at the age of 5–11 months.

## Introduction

Articular cartilage (AC) is a specialized connective tissue covering the ends of articulating bones within the joints. Main constituents of AC are a network of fibrillar collagen, proteoglycans (PGs), interstitial water and chondrocytes^1,2^. Adult AC can be divided into three zones (superficial, middle and deep) based on the orientation of the collagen fibrils^2,3^. In the relatively thin superficial zone, thin collagen fibrils run parallel with the articular surface and the PG content is low^4^. In the middle zone, the collagen fibrils are randomly oriented, while in the deep zone they run perpendicular to the articulating surface. The PG and collagen contents are high and the fibrils become thicker in the deep zone^4–6^. The main tasks of AC are to provide almost frictionless movement in joints and to distribute and mitigate loads generated by locomotion. In compression, the AC distributes the loads while at the same time it has to be strong in tension resisting lateral expansion and providing integrity to the tissue^7,8^.

The tensile biomechanical properties of AC are provided by the specific structure of the collagen fibril network and the biochemical composition of the tissue^9–11^. The variations in AC tensile biomechanical properties can be attributed to the orientation of the collagen fibrils and differences in the amount and spatial distribution of macromolecules, such as collagen and PGs^12^. During growth and maturation, the distribution of the AC constituents alters significantly^13^. In AC of a newborn mammal, the tissue appears structurally homogeneous and the collagen fibril network is not yet organized in a zonal manner^10,13,14^. The collagen network orientation gradually develops into the typical inhomogeneous Benninghoff architecture during maturation of the tissue^15–18^.

The changes in the collagen network orientation and the composition of AC during maturation and growth have been previously studied, but there is limited knowledge of the biomechanical changes arising from growth and development^10,13,14,19,20^. To understand the composition-structure-function relationships in mature and immature AC, the above-mentioned age-dependent alterations are of great importance. Furthermore, information about the alterations in AC composition during growth may be useful when conceiving means to prevent osteoarthritis (OA), because the remodelling capacity of the mature AC is dramatically low, practically absent^21^.

The spatial distribution of macromolecules within AC, not detectable with conventional biochemical methods, can be revealed with hyperspectral imaging^12,19,22–24^. Fourier transform infrared microspectroscopy (FTIR-MS) is a non-destructive, non-invasive and label-free imaging method^25^. With FTIR-MS, absorption spectra from the sample are collected in a pixel-by-pixel fashion, forming a hyperspectral image, where each spatial coordinate has its own absorption spectrum. The simplest way to gain information from these spectra is by using univariate methods. For example, collagen or PG contents in AC can be approximated from the absorbance of the amide I and the carbohydrate regions, respectively^22,24^. Alternatively, partial least squares regression (PLSR) of the spectra can be used as a more complete descriptor of the biochemical composition of AC. For example, we have earlier used PLSR to predict the compressive biomechanical properties of bovine AC from FTIR-MS data^26,27^.

The biochemical composition and structure of AC affect the biomechanical properties of the tissue, and the compositional variations are related to joint loading^11,12,28–30^. It has been suggested that the tensile biomechanical properties of AC are primarily controlled by the collagen network^12,31,32^. The relationship between the tissue composition, collagen fibril network arrangement, and tensile properties can actually be very complex in different phases of tissue maturation. The first aim of this study was to investigate, using FTIR-MS and polarized light microscopy (PLM), how AC composition and structure change during maturation. The second aim of the study was to determine the relationships between the AC composition, arrangement of the collagen network, and tensile biomechanical properties. We hypothesized that during maturation, arrangement of the collagen network and the collagen content of the AC affect profoundly its tensile biomechanical properties.

## Results

### Biomechanics, composition and structure

**Figure 1:**
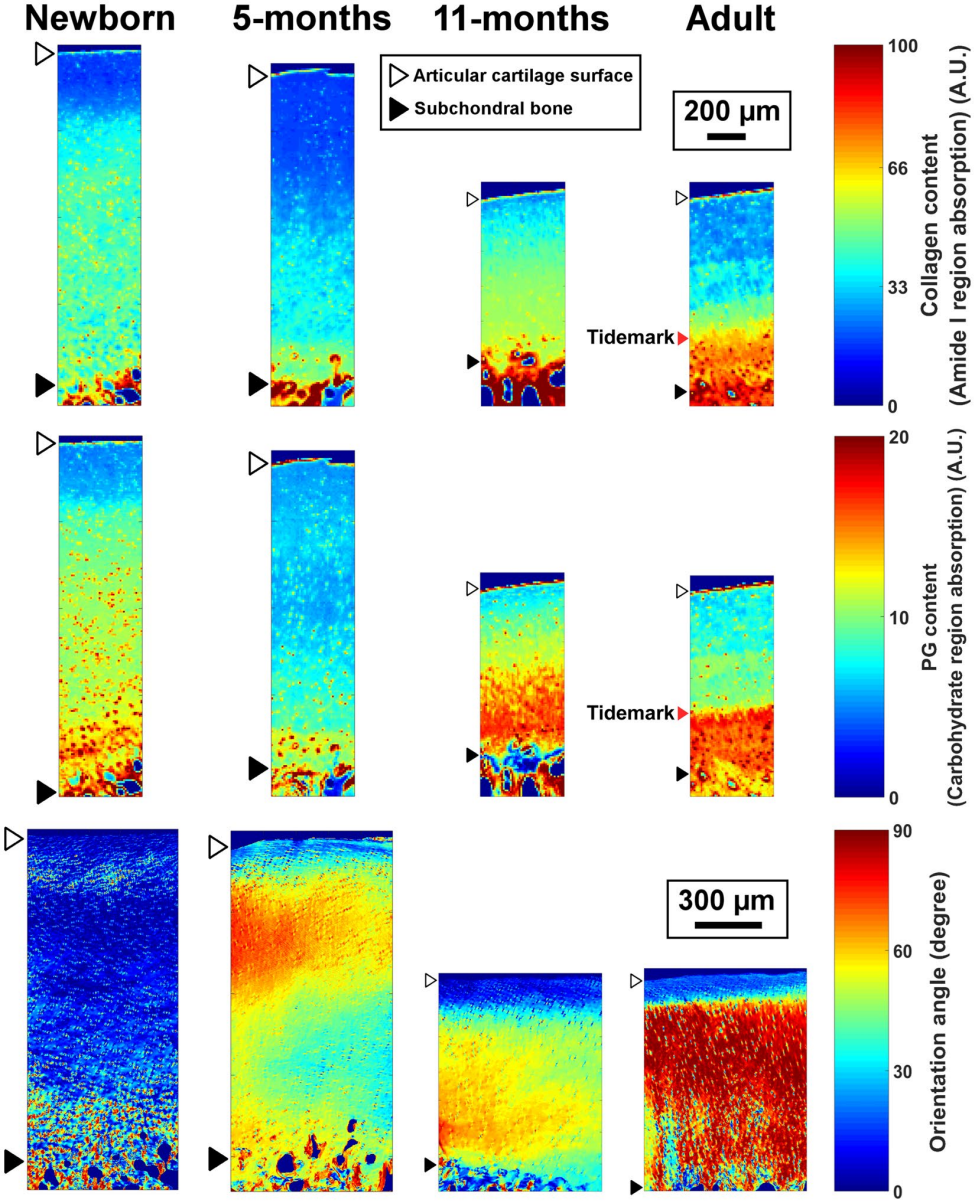
Equine articular cartilage development during growth and maturation. Upper row: The distribution of collagen content (amide I absorption). Middle row: The distribution of PG content (carbohydrate region). Bottom row: The mean orientation angle of collagen fibril network obtained by PLM. The collagen fibrils with an orientation angle of 0° run parallel with the articular surface, whereas angle 90° corresponds to collagen fibrils running perpendicular to the articular surface. The tidemark was not clearly visible in the young groups.

**Figure 2:**
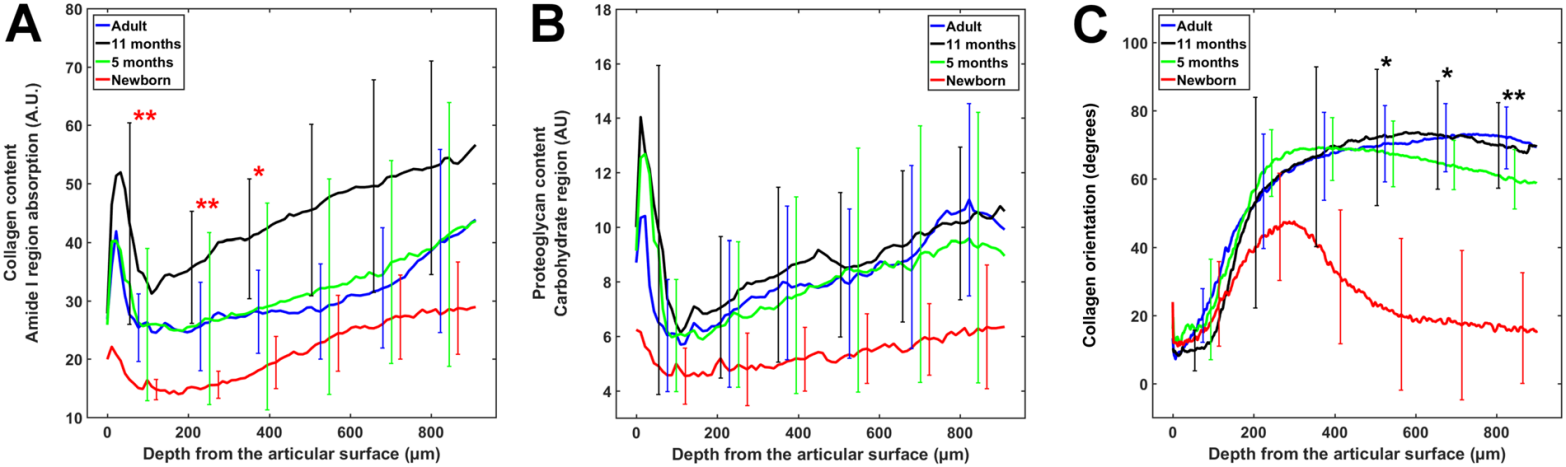
Distribution of collagen content (**A**) and proteoglycan content (**B**) according to the depth of articular cartilage in different age groups. Collagen content was estimated by the amide I region and proteoglycan content by the carbohydrate region. (**C**) Orientation of the collagen fibril network as a function of depth of the articular cartilage. In the plots, black stars indicate the difference between the adult and newborn groups, red stars indicate the difference between the 11-month-old and the newborn groups. *p < 0.05, **p < 0.01. A.U. = Absorption unit.

Representative FTIR-MS-based estimates of the collagen (amide I region absorbance) and the PG (carbohydrate region absorbance) content distributions, as well as the collagen fibril network orientation (PLM images) are shown in Fig. 1. Profiles of the collagen and PG content, and the collagen fibril orientation with respect to cartilage depth are shown for each age group in Fig. 2. The overall collagen and PG contents were low in the newborn group and higher in the more mature groups. However, the only statistically significant (*p* < 0.05) difference was observed in the superficial collagen content between the 11-month-old and newborn animals (Fig. 2A). Use of second derivative peaks as indicators of the collagen and PG content resulted in similar depth-wise profiles for the groups (see Supplementary materials), but also the difference between the adults and newborns in the collagen content reached statistical significance (*p* < 0.05). The collagen fibril orientation was significantly different in the middle and the deep layers between the adults and newborns (Fig. 2C). However, the collagen fibril orientation in the superficial and middle zones of the cartilage resembled that of mature tissue (Benninghoff-type orientation) already in the 5-month-old animals (Fig. 2C).

**Figure 3:**
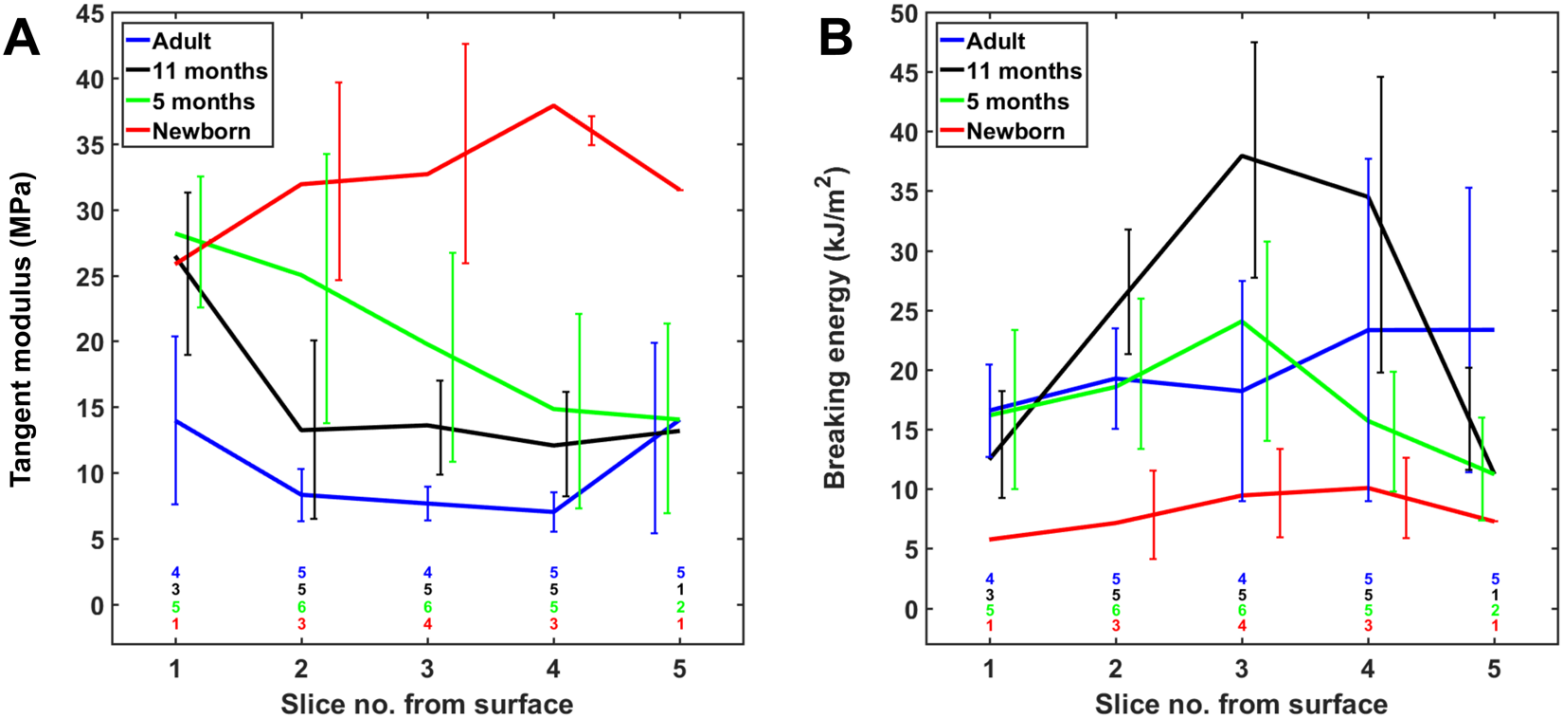
Profiles of the biomechanical parameters of cartilage sections according to the tissue depth (from 155 µm up to 930 µm) for the (**A**) tangent modulus and (**B**) breaking energy. Statistical comparisons between the age groups were not conducted because of the low number of samples at some depths. Colored numbers indicate the number of tested sections in each age group.

The tangent modulus and breaking energy of the tissue sections taken from different depths of AC are shown for each age group in Fig. 3. In general, the tangent modulus seemed to decrease with growth and maturation, especially in the deeper layers. In contrast, the energy required to rupture cartilage in tension seemed to increase after birth. Statistical comparisons between the groups at different cartilage depths could not be performed due to low number of samples (see the limitations of this study in the Discussion section).

**Figure 4:**
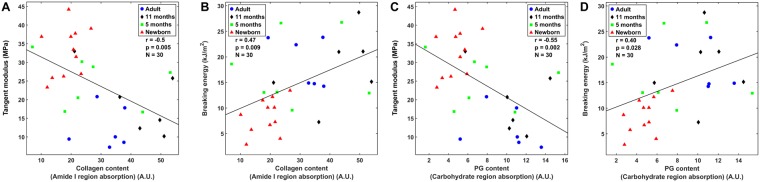
Correlation analysis between the collagen content and the tangent modulus (**A**) and between the collagen content and the breaking energy. (**B**) Data from samples with collagen orientation angles between 0 and 40 degrees from all age groups was pooled. Corresponding correlation analysis between the proteoglycan content and the tangent modulus (**C**) or proteoglycan content and the breaking energy. (**D**) A.U. = Absorption unit.

Statistically significant correlations were observed between the collagen content and the tangent modulus (*r* = −0.50, *p* = 0.005, 95% CI [−0.73, −0.17]) (Fig. 4A), the collagen content and the breaking energy (*r* = 0.47, *p* = 0.009, 95% CI [0.13, 0.71]) (Fig. 4B), the PG content and the tangent modulus (*r* = −0.55, *p* = 0.002, 95% CI [−0.76, −0.23]) (Fig. 4C), and the PG content and the breaking energy (*r* = 0.40, *p* = 0.028, 95% CI [−0.05, −0.67]) (Fig. 4D). Similar correlations were obtained with the second derivative parameters (see Supplementary Material).

**Figure 5:**
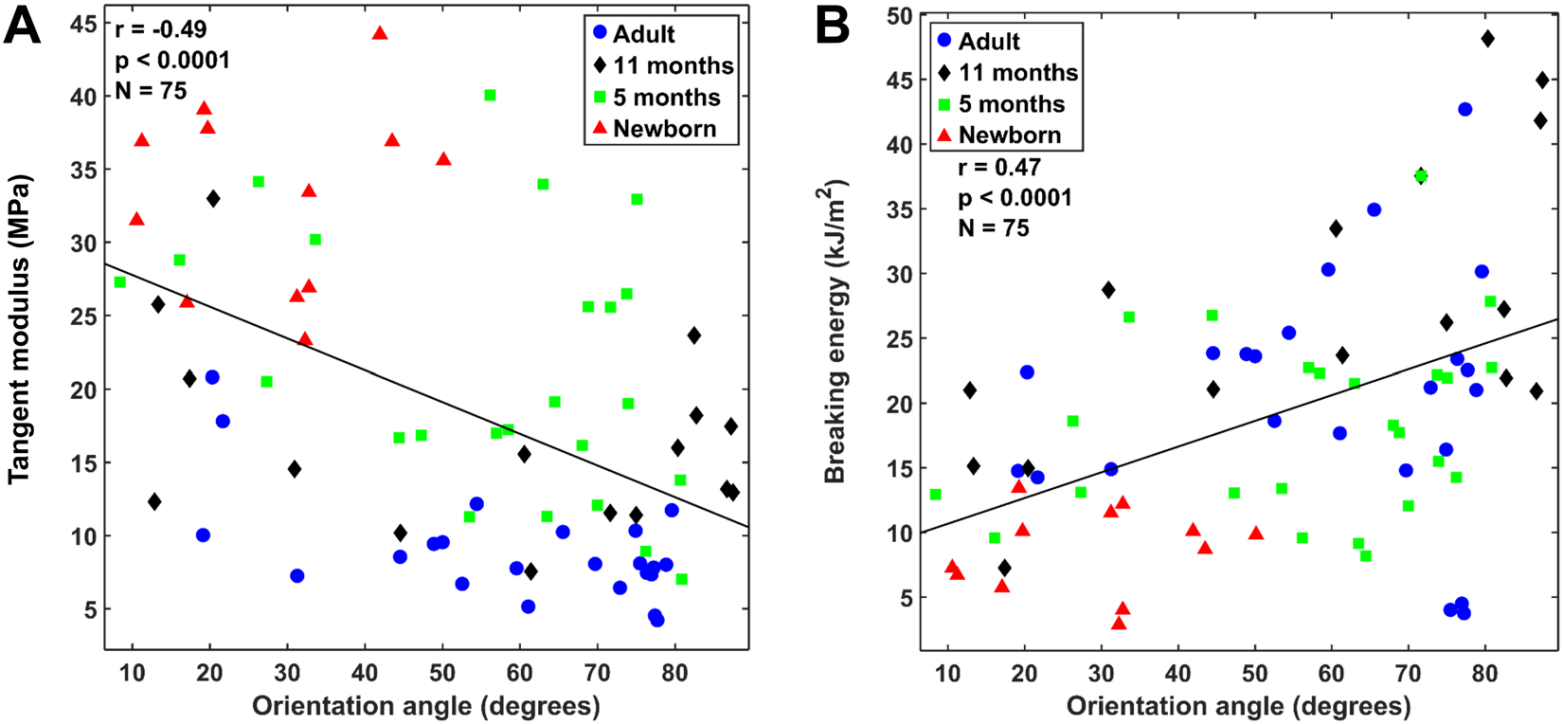
Correlation analysis between the orientation angle of the collagen fibril network (obtained by PLM) and the tangent modulus (**A**) and between the orientation of the collagen fibril network and the breaking energy. (**B**) The correlation analysis was conducted between the average orientation angle value of each depth and the corresponding biomechanical parameters from all age groups.

There was a significant negative correlation between the collagen fibril orientation angle and the tangent modulus (*r* = −0.49, *p* < 0.001, 95% CI [−0.30, −0.65]) (Fig. 5A), while a positive correlation was seen between the collagen fibril orientation angle and the breaking energy (*r* = 0.47, *p* < 0.001, 95% CI [0.63, 0.27]) (Fig. 5B). In this analysis, the collagen fibrils with an orientation angle of 0° run parallel with the articular surface, whereas fibrils with an angle of 90° run perpendicularly to the cartilage surface.

In addition to the simple regression analysis for pairwise comparisons, a multivariable linear regression model was used to predict the tensile modulus and the breaking energy based on the collagen orientation, collagen content and PG content (Table 1). The tensile modulus was predicted to be higher in the samples with higher collagen content, lower PG content and with fibril orientation parallel to tension (*R*^2^ = 0.382). On the other hand, the breaking energy was predicted to be higher in the samples with higher collagen content, higher PG content and fibril orientation perpendicular to tension (*R*^2^ = 0.376). In both models, all the predictors were significant (*p* < 0.05).

**Table 1:** Regression coefficient estimates (*B*) with standard errors (s.e.) for the linear multivariable regression models.

	Predictors (mean ± s.d.)	Model 1: Tensile modulus		Model 2: Breaking energy	
		*B*_*x*_ (±s.e.)	*p*-value	*B*_*x*_ (±s.e.)	*p*-value
*B*_0_	Constant	33.862 ± 2.829	0.160	2.895 ± 2.263	0.205
*B*_1_	Collagen orientation (53.1 ± 23.9)°	−0.168 ± 0.043	0.000	0.194 ± 0.042	0.000
*B*_2_	Collagen content (32.5 ± 16.5) Absorbance	0.481 ± 0.202	0.001	0.105 ± 0.031	0.002
*B*_3_	Proteoglycan content (9.1 ± 4.5) Absorbance	−2.431 ± 0.752	0.023	0.271 ± 0.117	0.024
Model performance		*R* = 0.618 *R*^2^ = 0.382	*p* < 0.001	*R* = 0.613 *R*^2^ = 0.376	*p* < 0.001

Models for both the tensile modulus (Model 1) and the breaking energy (Model 2) had collagen orientation (ORI), collagen content (COL) and proteoglycan content (PG) as predictors. The regression equations were of form *Y* = *B*_0_ + *B*_1_ · ORI + *B*_2_ · COL + *B*_3_ · PG.

### Partial least squares regression - PLSR

**Figure 6:**
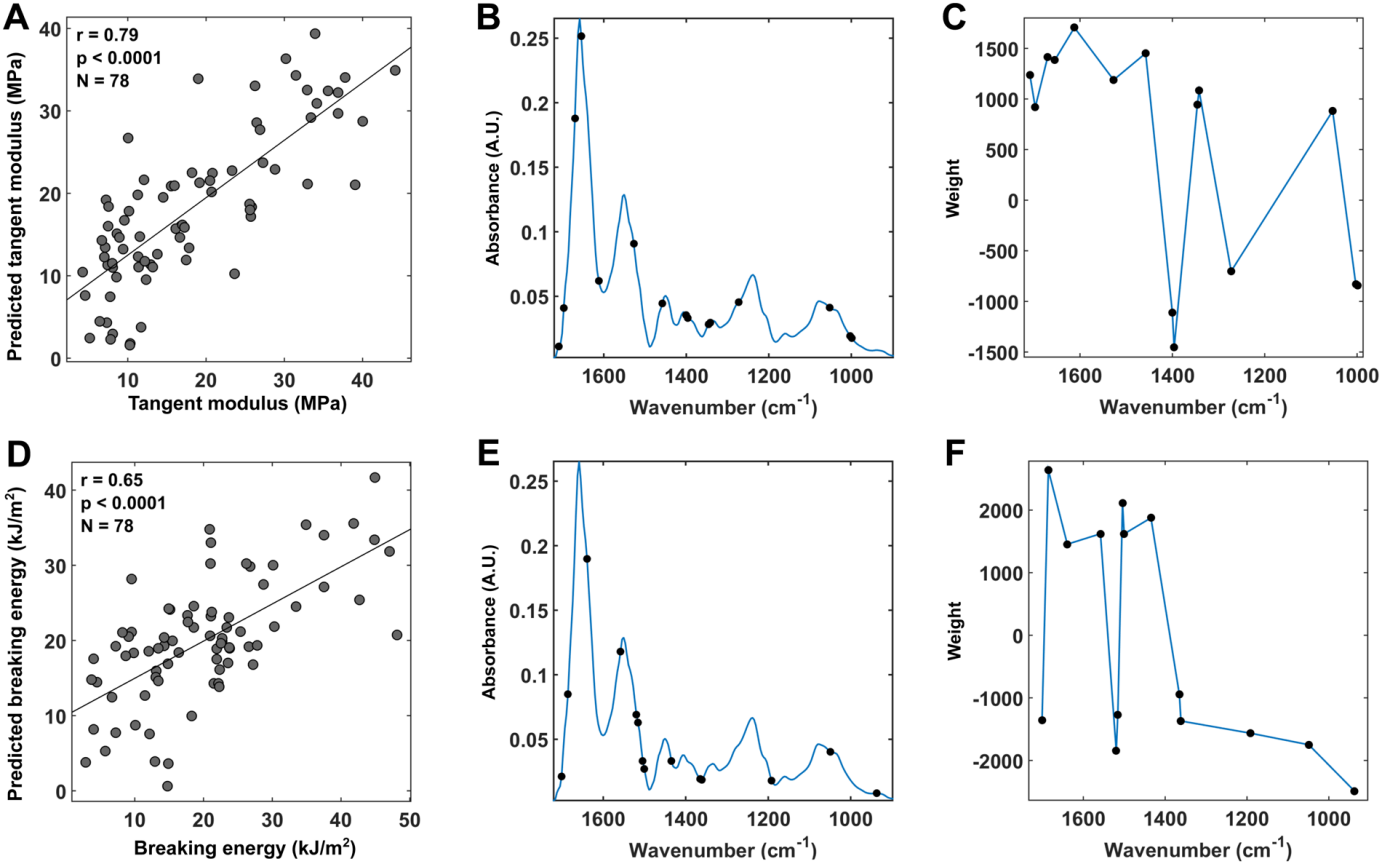
Measured values plotted against PLSR predictions for the tangent modulus (**A**) and the breaking energy (**D**). The values predicted by the PLSR are shown for wavenumbers selected by the CARS. The wavenumbers selected by the CARS are marked with black dots on the average spectra of both models (**B** and **E**). The weights of each selected wavenumber are shown for both models (**C** and **F**).

The PLSR was used to find out how well the FTIR-MS data, which represents the overall biochemical composition of the tissue, can explain the tensile properties of articular cartilage. The PLSR analysis combined with the competitive adaptive reweighted sampling (CARS) algorithm was significantly correlated with the tensile modulus (*r*_*E*_ = 0.79, *p* < 0.0001, 95% CI [0.69, 0.86]) and the breaking energy (*r*_*W*_ = 0.65, *p* < 0.0001, 95% CI [0.50, 0.76]) of AC (Fig. 6A and D). The optimal number of latent variables (LVs) was nine for both models (the tangent modulus and the breaking energy). The CARS algorithm selected 15 wavenumbers as most important for prediction of the tangent modulus and 14 for the breaking energy. The selected wavenumbers are marked with black dots in the average spectra of the corresponding model (Fig. 6B and E). The prediction did not improve significantly when the second derivative spectra were used as predictors. When predicting the tangent modulus, the regression coefficients showed positive values at the amide I region, the amide II region and the region around the peaks 1338 cm^−1^ and 1062 cm^−1^ (Fig. 6C). The amide I and the amide II regions and peak 1338 cm^−1^ are suggested to arise primarily from collagen^23,24^. For the breaking energy, the prediction was less accurate (Fig. 6D). The regression coefficients (Fig. 6F) showed positive values around the amide I and the amide II regions, except around wavenumber 1520 cm^−1^.

## Discussion

Tensile testing, FTIR-MS and PLM were used to study the relationship between the composition, structure and tensile biomechanical properties of equine AC at multiple depths and different stages of growth and maturation. Significant structural and compositional changes in AC occurred during maturation, and these changes were related to the tensile biomechanical properties.

Comparison between the age groups shows that the net collagen and PG contents of AC increase during maturation (Fig. 2A and B). It seems that the collagen and PG contents increase gradually during the first year of maturation, but then slightly decrease, especially in terms of the collagen content. However, statistically the only difference was observed between the newborn and 11-month old animals. Increasing collagen content with maturation is consistent with previous studies^19,28,33–37^. In the literature, the PG content has been shown to increase^13,35^, decrease^19,33,36^ or remain unaltered during maturation^38,39^, making direct comparisons challenging. On the other hand, the collagen fibril network orientation rapidly developed into the arcade-like architecture (Figs 1 and 2). This typical Benninghoff^13^ structure was evident at the age of 5–11 months. Consistent with our data, it took around 40 weeks for the collagen network to develop into an adult-like orientation in ovine AC^10^. In addition, the collagen orientation profiles in the adult and 5–11-month old groups are similar to the collagen orientation profiles of mature equine AC acquired with small angle X-ray scattering^17^. Most remarkably, the immature cartilage appears to consist of laminar layers of collagen fibrils parallel to the surface, consistent with earlier studies^10,13,16,37^, which probably explains the high tensile stiffness of this age group through tissue depth (Fig. 3).

When considering the average values through the tissue depth, it seems that the tensile modulus decreases with age, whereas the breaking energy increases with age (Fig. 3). In the newborn group, the tangent modulus was high throughout the tissue depth (mean 33 MPa) and the breaking energy was low (mean 8.5 kJ/m^3^) (Fig. 3A and B). This is likely due to the combination of the collagen fibrils running parallel with the articular surface and the low collagen content through almost the whole tissue depth (Fig. 2). On the other hand, the mean tensile moduli through the cartilage depth were 21, 16 and 9 MPa for the 5-month-old, 11-month-old and adult cartilages, respectively, whereas the average values of the breaking energy were 18, 27 and 20 kJ/m^3^ for the same age groups. The age-related changes in the tensile modulus seem to relate to the development of the collagen fibril architecture; especially the orientation perpendicular to the cartilage surface in the deep layers is an indicator of the low tensile modulus in mature cartilage. The changes in the breaking energy, on the other hand, are likely related to the development of the collagen content after birth, such as can be seen from the high values in the 11-month-old group compared to the other groups.

Consistent with our tensile modulus values, previous studies on adult equine AC from similar joint locations have reported values for the tensile modulus from 8 to 14 MPa^40^ and from 10 to 28 MPa^41^, depending on the site, cartilage depth and direction of split lines. Furthermore, data on the compressive properties of equine AC seem to be consistent with the tensile modulus values as reported in the current study, as mean compressive dynamic modulus of equine AC from the same region as tested here was ~6.5 MPa for newborn foals and had decreased to approximately 3.5 MPa in adult horses^20^. Similarly with the tensile modulus, the dynamic compressive modulus is also mainly controlled by the collagen network (with fluid in compression)^37,42^. We are not aware of other studies that have tested newborn equine AC in tension, but the dynamic tensile modulus for fetal bovine AC from femoral condyles has been reported to be approximately 4 MPa^28^. On the other hand, the mean dynamic tensile modulus for both calf (1–3 weeks old) and adult (1–2 years old) bovine femoral AC was reported earlier to be approximately 12 MPa. The latter value is similar to the one for mature AC in the current study, but the value for fetal cartilage is lower. In addition, the failure strain was higher in fetal bovine AC compared to more mature species, whereas this is the other way around for our samples^28^. It is possible that these differences in results measured for immature cartilage are due to the differences in the studied species and anatomical locations (equine proximal phalanx and bovine femur), as the cartilages from these different locations can be subjected, and adapted, to different biomechanical environments during gestation. For example, fetal bovine AC is rich in proteoglycans, whereas our newborn cartilages had lower PG content compared to the more mature cartilages (Fig. 2). Further, it has recently been shown that there are inherent differences between the equine and bovine species in the development of both composition and architecture of subchondral trabecular bone^43^.

The breaking energy correlated significantly with the PG and collagen contents (Fig. 4 and Table 1), which is consistent with other studies^28,44^. Previous studies have reported positive correlations between the tensile stiffness and collagen content in human and bovine AC^28,45^. To our surprise, here the collagen content showed statistically significant negative correlations with the tensile modulus (Fig. 4A and C). However, in the multivariable regression model, the tensile modulus correlated positively and significantly (*p* < 0.01) with the collagen content (Table 1). This seems to reflect a situation where confounding factors (e.g., collagen orientation) may bias the results of the simple pairwise correlation analysis. Another confounding factor could be, for example, ultrastructural inter-connectivity of the collagen network or the amount of cross-links in the collagen network^46^. These cross-links typically increase with age and may contribute to the higher breaking energy in mature AC. Even though analysis of cross-links was not carried out in the present study, it was previously shown in equine AC that there is an age-related increase in hydroxylysylpyridinoline cross-links (the major covalently bound cross-link in articular cartilage), which starts at the age of 11 months^47^. On the other hand, in more mature cartilage, more collagen that is not oriented along the loading direction, especially in the deeper layers, might have increased the failure strain and the breaking energy due to slower and progressive straightening of the high amount of collagen fibrils during loading. Furthermore, in the previous studies, the correlation between the collagen content and the tensile modulus in human femur was only found in healthy AC, but not in osteoarthritic samples^11^. This could be due to varying alterations in collagen network structure during OA, which could mask the effects of collagen content on tensile properties. In addition, the biochemical measures were not correlated with the mechanical properties in fetal bovine AC^28^. Based on these observations, we suggest that the importance of the AC composition is substantial to the tensile breaking energy, while alterations in the collagen network architecture during maturation define primarily the tensile modulus.

The negative correlation in simple and multivariable linear regression between the PG content and the tensile modulus was surprising, since earlier studies have suggested that swelling pressure caused by PGs increases the tensile stress in the collagen fibrils, leading to higher tensile and dynamic modulus^48–50^. Also, insignificant correlations between the tensile stiffness and PG content in canine and human AC have been found^45,51^. However, our result is consistent with the weak negative correlation between these parameters reported for bovine AC^28^. Our result is also consistent with the observations that removal of sulphated glycosaminoglycans with chondroitinase ABC increased the tensile stiffness of cartilage *in vitro* immediately after the treatment^52,53^. Furthermore, removal of glycosaminoglycans from bovine AC taken from calves (1–3 weeks) and young adults (1–2 years) with guanidine-HCl increased the equilibrium and dynamic tensile moduli of AC, whereas a similar phenomenon was not observed in more aged human AC attained from teens (aged 15–17 years) or adults (37–39 years)^34^. The increase in tensile integrity in younger AC was postulated to arise from removal of glycosaminoglycans that may inhibit collagen network interactions, whereas in more aged AC the collagen network may have attained a state where such interactions cannot be anymore created^34^. This explanation could also be applicable here.

It has been postulated previously that the ratio between collagen and PG contents is more strongly correlated with the tensile modulus of cartilage than either parameter alone^11^. To test this, we also investigated this ratio by taking the amide I to carbohydrate region ratio from the spectra. The correlation between this ratio-parameter and biomechanical properties, however, was not significant (see Supplementary material). This may be caused by the fact that the collagen-to-PG ratio varies only little (see Supplementary material) as both the PG and collagen contents increase simultaneously during maturation of the equine AC (Fig. 2A and B).

The results from PLSR analysis of FTIR-MS data showed higher correlation to the biomechanical parameters compared to the univariate methods, supporting the idea that the tensile biomechanical properties of AC cannot be fully explained by the characteristics of a single structural component only. For both biomechanical parameters, the strongest regression coefficient was obtained within the amide I spectral region (Fig. 6C and F). Moreover, wavenumbers became selected from regions close to the carbohydrate region (1140–984 cm^−1^) and the amide II region (1580–1490 cm^−1^). Also wavenumbers from the vicinity of peaks 1400 cm^−1^, 1376 cm^−1^ and 1338 cm^−1^ were selected (clearly visible in the second derivative spectrum^54,55^). The amide I region (C=O stretch), amide II region (C-N stretch and N-H bend) and especially the peak 1338 cm^−1^ (CH_2_ side chain of collagen), which is commonly referred as “collagen integrity”, are suggested to arise primarily from the collagen network^23,24^. On the other hand, the lower wavenumbers in the carbohydrate region arise mainly from the sulphated sugars within PGs^56^ and the peaks 1400 cm^−1^ (COO^−^ stretch) and 1376 cm^−1^ (CH_3_ symmetric bend) arise from bond vibrations found in PGs^54,55^. Drawing definite conclusions about the importance of each distinguished wavenumber is challenging, but the strong regression coefficients in collagen-related wavenumber regions (i.e., Amide I, Amide II and 1338 cm^−1^) indicate that collagen is the most important regulator of the tensile properties. Consistent with the previous simple and multivariable regression analyses, wavenumbers related to the PGs were selected, indicating that also PG content is important for tensile properties, but only to some degree, as these achieved lower loadings in the PLSR model.

Using the amide I region of the FTIR spectrum to estimate the collagen content may be precarious, since it has been shown that the amide I region arises from the C–O stretching vibrations and this molecular bond can be found not only in the collagen, but also in other proteins^54,57,58^. For example, elastin has a very similar spectrum compared to collagen, and elastin has been suggested to form a functional structure in the superficial layer of AC^40^. Moreover, one of the most important glycosaminoglycans in the AC tissue, chondroitin sulphate, also significantly contributes to the amide I region. When comparing the FTIR-based collagen depth-wise distribution with the biochemical reference methods, there seems to be a disagreement with a previous study, in which a decrease of collagen content with cartilage depth in human femoral cartilage was reported^59^. However, in another study a similar collagen content distribution as seen in our study was reported for immature and fetal bovine cartilages, when measured with biochemical methods^36^. Furthermore, there are several FTIR studies showing the same depth-wise collagen content distribution as was observed here^4,35,60^. Hence, it seems that the distribution of AC constituents may vary between joint regions and species. Nevertheless, to better validate our results from the amide I region, we also calculated the second derivative parameters (from the peaks 1376 cm^−1^ and 1202 cm^−1^) in this study. The second derivative parameters, such as the peaks at 1376 cm^−1^ and 1202 cm^−1^, are more specific for PGs and collagen than the carbohydrate and amide I peak areas, respectively^54^. This is due to the fact that the second derivate spectra of these individual compounds do not overlap^58^. The second derivative parameters of the FTIR spectra supported our findings, as the results were very similar to the results obtained using the amide I and the carbohydrate region parameters for collagen and PGs, respectively (see Supplementary Material).

There are limitations to the present study. First, a basic concern regarding the use of the tissue sections in this study was that a few of the fathers of the foals (stallions) had equine osteochondrosis (OC), which is common in warmblood breeds. OC disturbs endochondral ossification and manifests as focal necrotic areas, detachment of cartilage flaps and formation of loose fragments^61^. Of course, this will affect the properties of the joint cartilage in affected areas. However, OC is a disorder that typically becomes manifest in well-defined predilection areas and does not affect articular cartilage in a systemic way. Because of this, all animals were checked for macroscopic evidence of OC in the studied joint and discarded from the study, if necessary. Second, the number of horizontal sections in each age group, taken at different cartilage depths, was limited. Especially sections taken from the superficial cartilage of the newborn group were often ragged or had holes, which may be remnants of vascularization. These samples were discarded for mechanical testing, which limited the available number of samples for these tests. For this reason, it was not possible to conduct statistical group comparisons of mechanical parameters between the different age groups. However, the sample size was sufficient for the microscopy and correlation analysis parts of the study. Third, specimens of the present study were stored in the freezer at −20 °C before collection of the specimens for microscopy and tensile testing. This may have affected the properties of AC. Some previous studies suggest that the mechanical compressive properties of AC are not affected by one freeze-thaw cycle at this temperature^62,63^, whereas some other studies have observed the opposite effect^64,65^. Ultimately, this limitation had to be accepted due to logistic reasons and must be taken in consideration. Fourth, we did not determine the split line direction of our samples. However, the tensile testing direction was always constant and a previous study suggests that the split lines run approximately perpendicular to this direction at the given joint region^40^. On the other hand, the split line direction should substantially affect only the first 155 µm-thick sections, since the contribution of the split lines to the anisotropy of tensile stiffness is higher in the superficial zone of cartilage^41,66^. However, it is poorly understood how split lines develop during maturation, and this should be studied in the future, for example, by investigating if split lines are present and generated in the deeper layers of immature cartilage where we see the fibril orientation to be parallel to the cartilage surface. Finally, the tissue response in tension is dependent on the used extension rate, which was 5 mm/min. This rate is consistent with many previous studies^28,51,67,68^. However, pure equilibrium properties of the solid matrix were likely not measured mainly due to collagen viscoelasticity^69^. On the other hand, since PGs are the major cause for Donnan osmotic pressure in AC, we cannot rule out the possibility that fluid pressurization due to osmotic pressure had some effect on the tensile mechanical properties at this extension rate. In addition, we cannot fully eliminate the effect of extension rate on the differences between the age groups.

We conclude that the composition and structure of equine AC undergo significant changes during growth and maturation, and that these changes manifest as altered tensile properties. Based on our findings, equine AC reaches the structure and composition resembling mature AC at the age of 5–11 months. The mature horses seem to have a less stiff collagen network in tension than newborns at this given region of the metacarpophalangeal joint, especially in the deep layers, but the collagen network is able to sustain higher loads and strains before failure, indicated by the greater breaking energy. It is suggested that these mechanical properties are mainly related to the collagen fibril orientation and collagen content, respectively. Prediction accuracy of the tensile properties during maturation and growth was moderate (*r* ~ 0.4–0.5) using univariate methods to analyze FTIR-MS data, whereas stronger (*r* > 0.7) correlations could be attained with partial least squares regression, demonstrating the usefulness of multivariate regression methods over simple regression.

## Materials and Methods

### Specimen preparation

**Figure 7:**
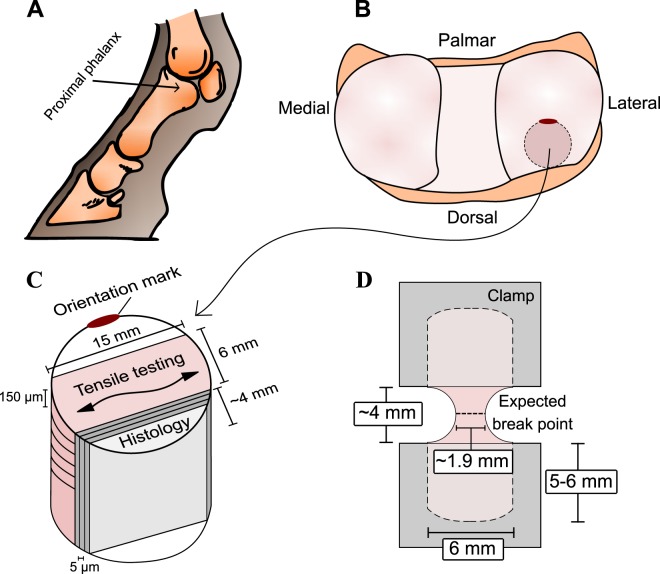
(**A**) Schematic figure of the left equine metacarpophalangeal joint. (**B**) Joint surface of the proximal phalanx shows the sampling site of the osteochondral plug. An orientation mark was made with a pointed instrument on the osteochondral block. (**C**) This orientation mark was utilized in plug trimming and in section preparation to ensure that from each block the horizontal frozen sections for tensile testing were always cut according to the line determined by the frontal plane, i.e., along a line running in lateral to medial direction. (**D**) Positioning of a specimen between the clamps. The expected area of breaking is indicated.

Equine AC samples (*N* = 25) of four different maturation levels (newborn, *n* = 8; 5-month-old, *n* = 6; 11-month-old, *n* = 5; and adult (6–10 years), *n* = 6) were collected. The joints and the cartilage surfaces studied were deemed normal by radiologic, macroscopic and microscopic evaluation. The samples were originally collected for other studies with the permission from the ethical committees of Utrecht University, the Netherlands, and Massey University, New Zealand, and all the procedures were in accordance to the local laws and regulations^70^. Two sets of osteochondral samples from the metacarpophalangeal joint were prepared, one for the tensile testing and another one for the microscopic and spectroscopic measurements. Samples were taken from the dorsal aspect of the articular surface of the proximal phalanx, which is a location subject to intermittent peak loading (Fig. 7A and B)^29^. A cartilage-on-bone plug (diam. 15 mm) was drilled and separated from the underlying bone. Before freeing the plug from the bone, an orientation mark was made on the plug surface with a pointed instrument. Utilizing this orientation mark, the plug was trimmed to have en-face dimensions of 15 mm × 6 mm, with the long side of the rectangle running in lateral to medial direction (Fig. 7B and C). This allowed a consistent orientation of the horizontal tissue sections in tensile tests. Joint surface split line orientation was not determined in order to keep the cartilage surface intact. Coronal tissue sections for histology were prepared from adjacent areas of the same plug (Fig. 7C).

Horizontal frozen sections of the articular cartilage surface were cut from cartilage-on-bone blocks and decalcified with Na-EDTA. The blocks were embedded with O.C.T. embedding medium in Cryomold molds (size 25 mm × 20 mm × 5 mm, Tissue-Tek^™^). Frozen sections with an average thickness of 155 ± 37 µm were cut with a cryostat microtome (Reichert-Jung Frigocut 2800, Nussloch, Germany) through the whole depth of the AC. Individual sections were stored in cryogenic vials containing isotonic saline solution (0.9% sodium chloride) with proteinase inhibitors (2 mM EDTA, 5 mM benzamidine HCL, 10 mM N-ethylmaleimide, pH 7.0–7.5). This solution was also used to moisten the specimens during tensile tests. Sections that were ragged and/or included holes were excluded from the sample set. Section thickness was measured with a contact sensing digital micrometer (Mitutuyo 0–25 mm). The gauge area was made with a plexi-glass mould, which had two holes for a 4-mm-diameter biopsy punch (see Fig. 7D). The average gauge width was 1860 µm. The gauge width of cartilage was measured with a computer-assisted stereomicroscope (Leica MX75 stereomicroscope, Leica Microsystem Ltd., Heerbrugg, Switzerland; Nikon Camera Head DS-5M and Camera Control Unit DS-L1, Nikon Corporation, Tokyo, Japan). The sections ready for tensile tests were stored in the freezer at −20 °C. Prior to tensile tests, the specimens were thawed at room temperature and laid on an ice bath until testing.

For polarized light microscopy (PLM) and the FTIR-MS, the cartilage-on-bone blocks were fixed in 10% formalin solution for 48 h and decalcified with Na-EDTA before embedding in paraffin. After cutting the histological sections (thickness = 5 µm), paraffin was removed with xylene. The sections for PLM were treated with hyaluronidase for 18 h to remove proteoglycans. The unstained sections for PLM were taken on standard objective glasses and covered with D.P.X. (Difco, East Molesey, UK) and coverslips^71^. For FTIR-MS, the sections were placed on 2-mm-thick ZnSe windows^26^.

### Tensile testing

Tensile testing was carried out using a Lloyd LFPlus mechanical testing instrument (Lloyd Instruments, Inc., Amtec, Paoli, PA, USA). Five rectangular sections from different AC depths were tested per animal. A borderline of five sections per animal was chosen as subchondral bone was frequently observed at greater AC depths based on our preliminary data. The mechanical data were successfully acquired from 78 sample sections (*N*_*adult*_ = 23, *N*_11 *months*_ = 19, *N*_5 *months*_ = 24, *N*_*newborn*_ = 12). During the test, the gauge area of the cartilage section was set between the two clamps (Fig. 7D). The sandglass shape of the sections guaranteed a consistent location of the ruptures (compared to more traditional dumbbell shape or rectangular with constant width); moreover, this shape prevents undesirable slipping at the clamps during testing, similarly to traditional dumbbell shaped samples. However, the drawback of this geometry is that the cross-sectional area will vary and calculation of stress will depend on the chosen location. We used the cross-sectional area from the thinnest point of the sample in our calculations, which will result in over-estimation of the modulus compared with, for example, the modulus calculated using the average cross-sectional area of the sample or with other measurement geometry (rectangular, dumbbell-shape). This should be kept in mind when comparing our results with previous studies with different sample geometries.

All specimens were tested with the static pulling speed of 5 mm/min, with 0.05 N preload. Clamp to clamp distance at preload was taken as initial sample length. The data, including time, force and clamp displacement were recorded continuously during the tests. From this data, the stress-strain curves were calculated. The slope from the linear region of the stress-strain curve (change in stress/change in strain) was used to determine the tangent modulus (*E*). The energy (*W*) needed to break the cartilage was calculated from the stress-strain curve by numerically integrating the area under the curve.

### Polarized light microscopy (PLM)

A conventional light microscope (Nikon Diaphot TMD, Nikon Inc., Shinaqwa, Tokyo, Japan) equipped with Abrio PLM system (CRi, Inc., Woburn, MA, USA) was used to study the orientation of the collagen fibrils^72^. Orientation images covering the full thickness of AC were acquired. Depth-wise orientation profiles were calculated from each section. The orientation images were repartitioned in 155-µm-thick sections to correspond with the sections used for tensile testing. The average value of this section was used in the analysis.

### Fourier transform infrared microspectroscopy (FTIR-MS)

Spectral data was acquired with an FTIR-spectrometer (Tensor 27, Bruker Inc., Billerica, MA, USA) coupled with a Bruker microscope (Hyperion 3000, Bruker Inc., Billerica, MA, USA) equipped with a 64 × 64 focal plane array (FPA) detector. Spectral resolution of 8 cm^−1^ and 64 repeated scans were used to image a 520 µm wide region from the subchondral bone to the surface of the AC. Pixel binning was used to set the pixel size to 21.6 µm.

A Resonant Mie Scattering Correction (RMieSC) algorithm was used for removing scattering effects from the spectral data^34^. Each hyperspectral data set was repartitioned in 155 µm thick sections to correspond with the locations of biomechanical measurements. Spectral regions of 1585–1720 cm^−1^ and 985–1140 cm^−1^ were used to estimate the collagen and the PG contents, respectively^24^. Earlier literature suggests that the second derivative parameters may be more specific for estimating collagen and PGs^54^. Therefore, to confirm our results of the collagen and the PG contents, the second derivative peaks 1202 cm^−1^ and 1376 cm^−1^ were calculated as additional estimates of the collagen and the PGs, respectively. Furthermore, the average spectrum of each 520 µm × 150 µm section was truncated to the spectral region of 900 cm^−1^ to 1720 cm^−1^ and vector-normalized for the PLSR analysis.

### Variable selection in FTIR-MS

Competitive adaptive reweighted sampling (CARS) is a novel method proposed by Li *et al*.^73^ to select an optimal combination of spectral variables (wavenumbers for the PLSR model. This method employs the “survival of the fittest” principle to select optimal variables based on their absolute regression coefficients to describe the phenomenon under investigation. A detailed description of the method can be found in reference^73^. Briefly, the CARS method works in four steps: (1) Monte Carlo for model sampling, (2) Exponentially decreasing function is used to define the ratio of preserved variables in each sampling run, (3) Competitive selection is realized with adaptive reweighted sampling, and finally, (4) the cross validation is utilized to select the subset of variables with the lowest root mean square error of cross validation (RMSECV).

### Partial least squares regression (PLSR) in FTIR-MS

The CARS algorithm was used to select the optimal wavenumbers for the PLSR model from the spectral region from 900 cm^−1^ to 1720 cm^−1^. The number of latent variables (LV) is selected using the leave-one-out cross validation approach. The optimal number of LVs is found when RMSECV reaches the global minimum. In the present study, we utilized ten-fold cross-validation, in which the sample set is divided into ten groups. Each group is removed at its turn from the data set and used for validation.

### Statistical analysis

The Pearson’s correlation analysis was used to study the relationships between the biomechanical and compositional parameters and between the collagen fibril orientation and the biomechanical parameters. Normality of variables was tested with the Shapiro-Wilk test and visually inspected from histograms and Q-Q plots. Earlier it has been shown that the orientation of the collagen network affects the biomechanical parameters^31^. Therefore, only the sample sections where the average orientation of the collagen network was between 0° and 40° were included in the simple regression analysis between biomechanical and compositional parameters to avoid a potential confounding effect of the collagen orientation. The degree of articular surface collagen fibril network orientation is 0°. Furthermore, multivariable linear regression models were built to predict the tensile modulus and breaking energy based on the collagen content, PG content and collagen orientation. In the PLSR prediction, simple linear regression between the predicted values and the reference values (tangent modulus and breaking energy) was used to evaluate the performance of the model. A confidence interval of 95% was used for data presentation. In the group comparisons, based on the Shapiro-Wilk test and visual inspection of data (histograms and Q-Q plots), normality could not be assumed for data from each group. Hence, the nonparametric Kruskal-Wallis test was used to analyze the pairwise differences between age groups. All spectral data processing was conducted with custom-made MATLAB scripts (R2015a, MathWorks Inc., MA, USA).

### Data availability statement

The datasets generated and analysed during the current study are available from the corresponding author on reasonable request.

## Electronic supplementary material

Supplementary materials
