# Supplementary material for: Composition, structure and tensile biomechanical properties of equine articular cartilage during growth and maturation
Source: Sci Rep. 2018 Jul 27;8:11357. doi: 10.1038/s41598-018-29655-5 (PMC6063957; doi:10.1038/s41598-018-29655-5)
Supplement: Supplementary file 1 — Supplementary materials [file 41598_2018_29655_MOESM1_ESM.pdf]

Composition, structure and tensile biomechanical properties of equine articular cartilage during growth and maturation

Oinas J., Ronkainen A.P., Rieppo L., Finnilä M.A.J., Iivarinen J.T., van Weeren R., Helminen H.J., Brama P.A., Korhonen R.K., Saarakkala S.

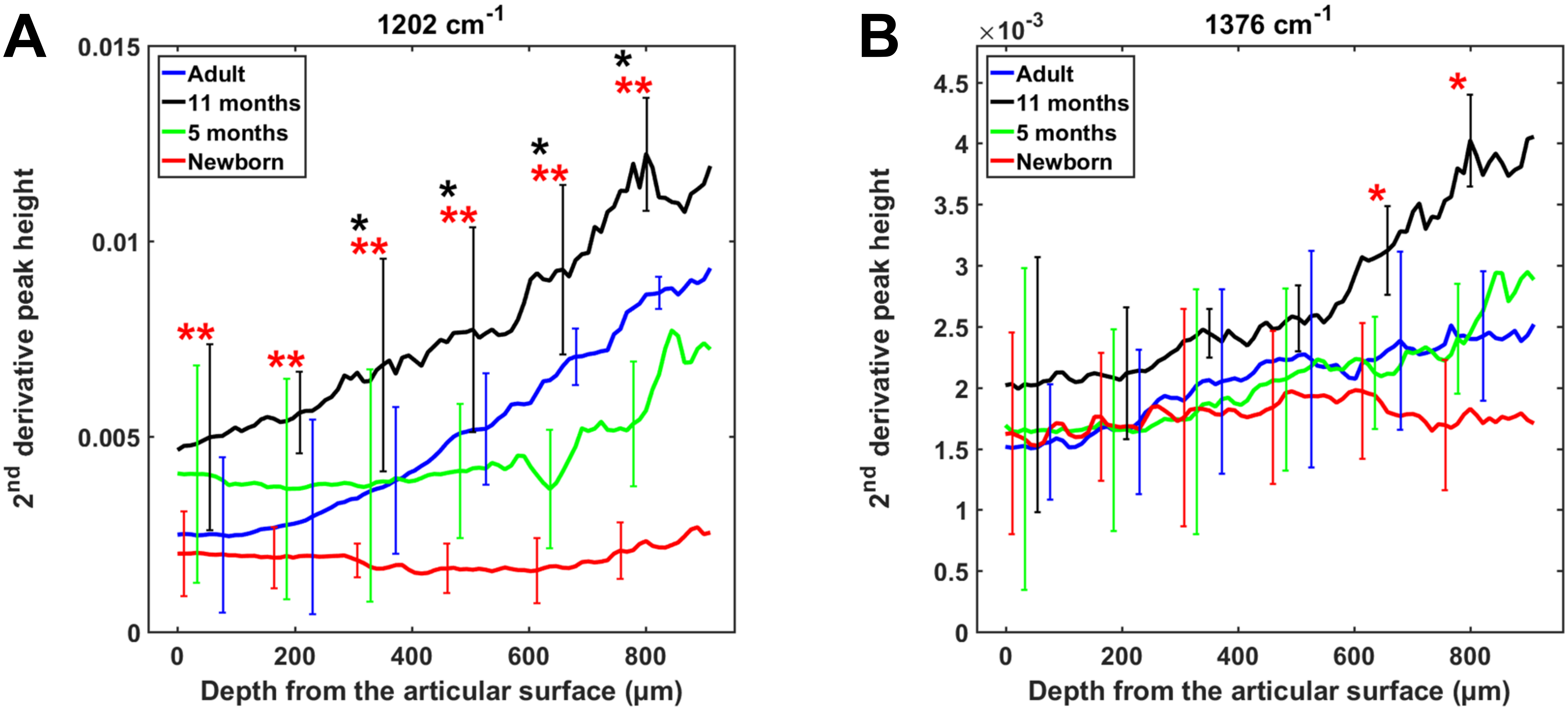

Supplemental figure 1: (A) collagen content estimated by the second derivative peak 1202 cm^{-1} and (B) PG content estimated by the second derivative peak 1376 cm^{-1} as a function of cartilage depth for each group. Black stars indicate a significant difference between the adult and newborn group, whereas red stars indicate a significant difference between the 11-month-old and newborn group. $^{*}p < 0.05$, $^{**}p < 0.01$.

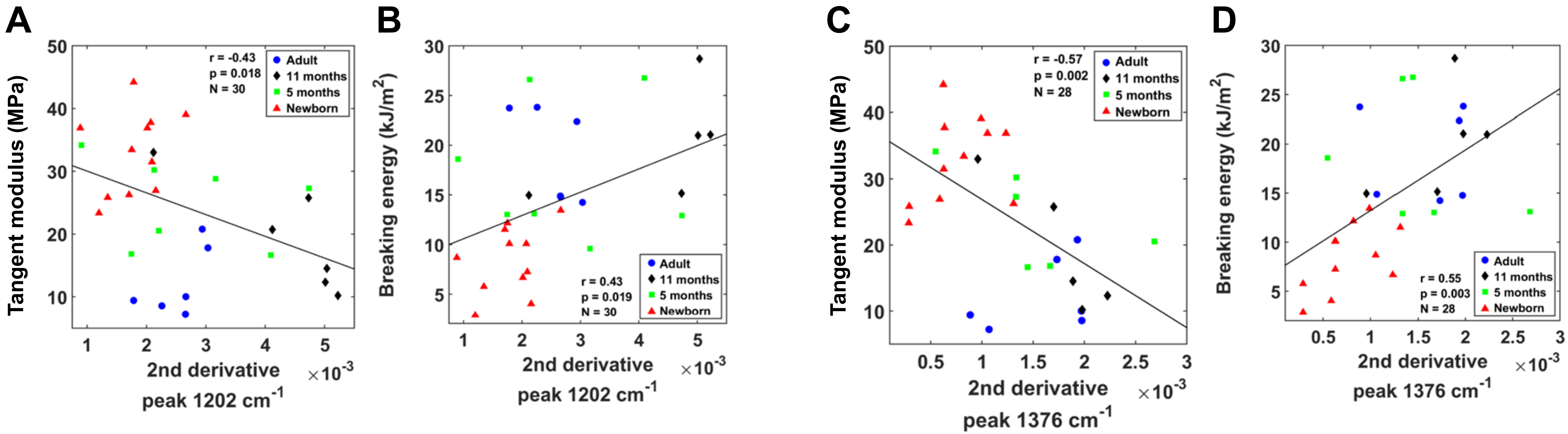

Supplemental figure 2: Correlation analysis between the second derivative peak 1202 cm-1 and (A) the tangent modulus and (B) the breaking energy. Corresponding correlation analysis between the second derivative peak 1376 cm-1 and (C) the tangent modulus and (D) the breaking energy.

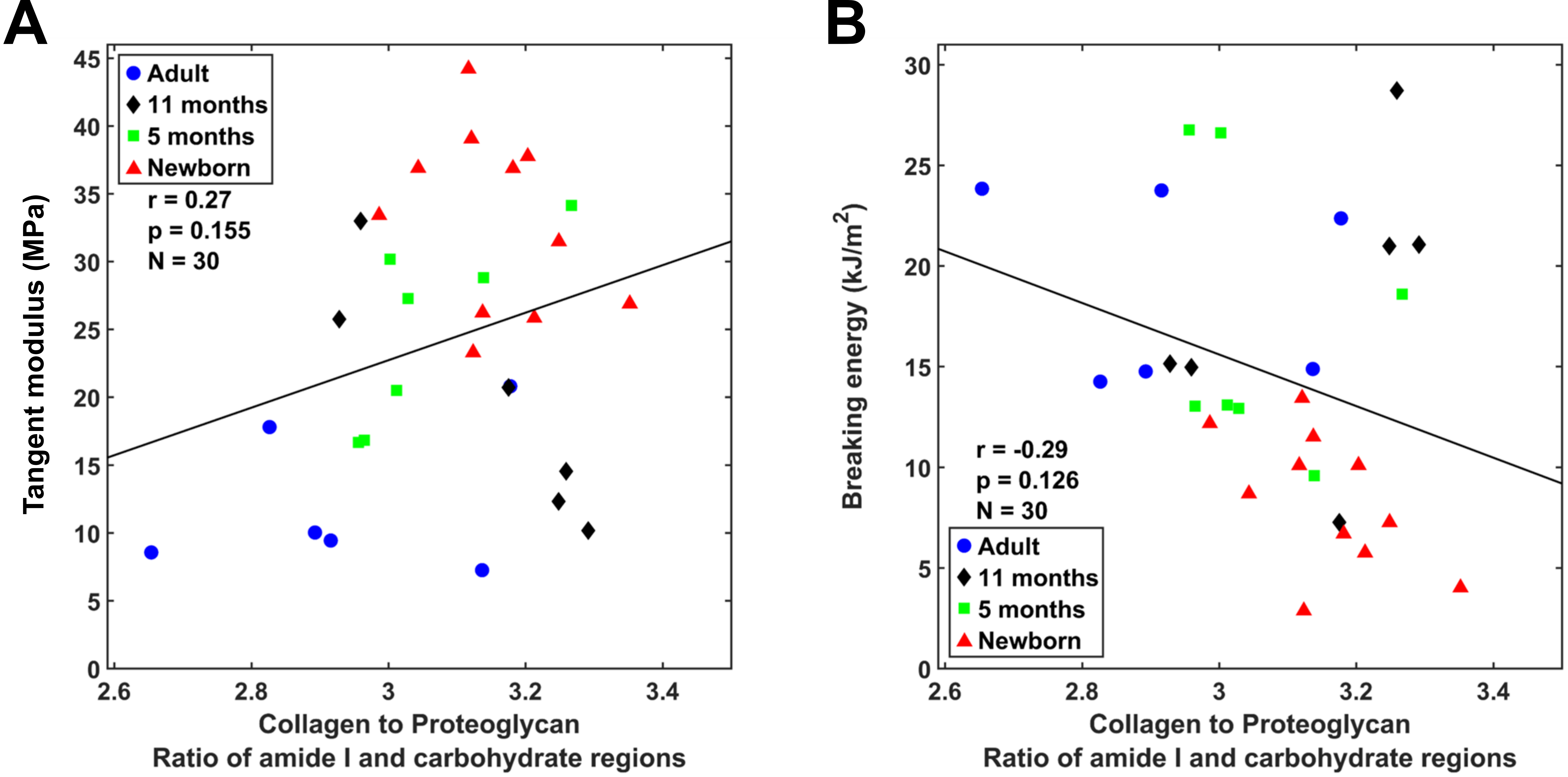

Supplemental figure 3: Correlation analysis between the ratio parameter (the amide I to the carbohydrate region) and (A) the tangent modulus and (B) the breaking energy.
